# Total Mercury, Methylmercury, Inorganic Arsenic and Other Elements in Meat from Minke Whale (*Balaenoptera acutorostrata*) from the North East Atlantic Ocean

**DOI:** 10.1007/s00128-017-2106-6

**Published:** 2017-06-02

**Authors:** Amund Maage, Bente M. Nilsen, Kaare Julshamn, Livar Frøyland, Stig Valdersnes

**Affiliations:** National Institute of Nutrition and Seafood Research, P. O. Box 2029, Nordnes, 5817 Bergen, Norway

**Keywords:** Minke whale, Mercury, Barents Sea, Methylmercury, Inorganic arsenic, Selenium

## Abstract

Meat samples of 84 minke whales (*Balaenoptera acutorostrata*) mainly from the Barents Sea, collected between 1 May and 16 August 2011, were analyzed for total mercury, methylmercury, cadmium, lead, total arsenic, inorganic arsenic and selenium. The average total mercury concentration found was 0.15 ± 0.09 mg/kg, with a range from 0.05 to 0.49 mg/kg. The molar ratio of selenium to mercury varied between 1.0 and 10.3. Cadmium content ranged from 0.002 to 0.036 mg/kg, while the content of lead in whale meat ranged from <0.01 to 0.09 mg/kg. None of the whale samples exceeded established EU maximum levels for metals in fish muscle, but 4.8% and 6.8% of the samples exceeded Japanese maximum levels for total mercury and methylmercury, respectively, in whale meat. There was only minor variations in element concentrations between whales from different geographical areas, and cadmium was the only element were the concentration increased with increasing length.

Even though mercury (Hg) is a naturally occurring element, it is regarded as a global pollutant that can impair both wildlife and human health. Of the different chemical forms of Hg, methylmercury (MeHg) is the most toxic and abundant in the marine food web. During the last several years, we have studied in detail the Hg content of two of the most important fish species in the Barents Sea, Atlantic cod (*Gadus morhua*) (Julshamn et al. 2013) and the Norwegian spring spawning herring (*Clupea harengus*), which spends its juvenile stage there (Frantzen et al. 2015). As the top predators are assumed to be most vulnerable to Hg accumulation in the Arctic (Scheuhammer et al. 2015), one can assume that whales are highly susceptible to Hg accumulation. The minke whale (*Balaenoptera acutorostrata*) in the North East Atlantic mainly forage on fish such as capelin, herring, mackerel and codfish and crustaceans such as krill (Haug et al. 2002; Pierce et al. 2004) in its main feeding seasons during spring and autumn. They eat less in the tropical breeding grounds (Morissette et al. 2010). The populations of minke whale in the North East Atlantic have been estimated by the International Whaling Commission (IWC) to contain between 64,000 and 100,000 whales over the last two decades (IWC 2016). There is still a limited hunting operation for the minke whale in the Arctic and some 1000 tons of whale meat have been put on the market annually in recent years (IMR 2016). We have studied total Hg (THg), MeHg, total arsenic (TAs) and inorganic arsenic (iAs) together with cadmium (Cd), lead (Pb) and selenium (Se) in whale meat from the 2011 hunting season to evaluate if there were differences in element concentrations with respect to sex of the animal or geographical area where the whales were caught, and whether there were any correlations between element concentrations and length of the animals.

## Materials and Methods

**Fig. 1:**
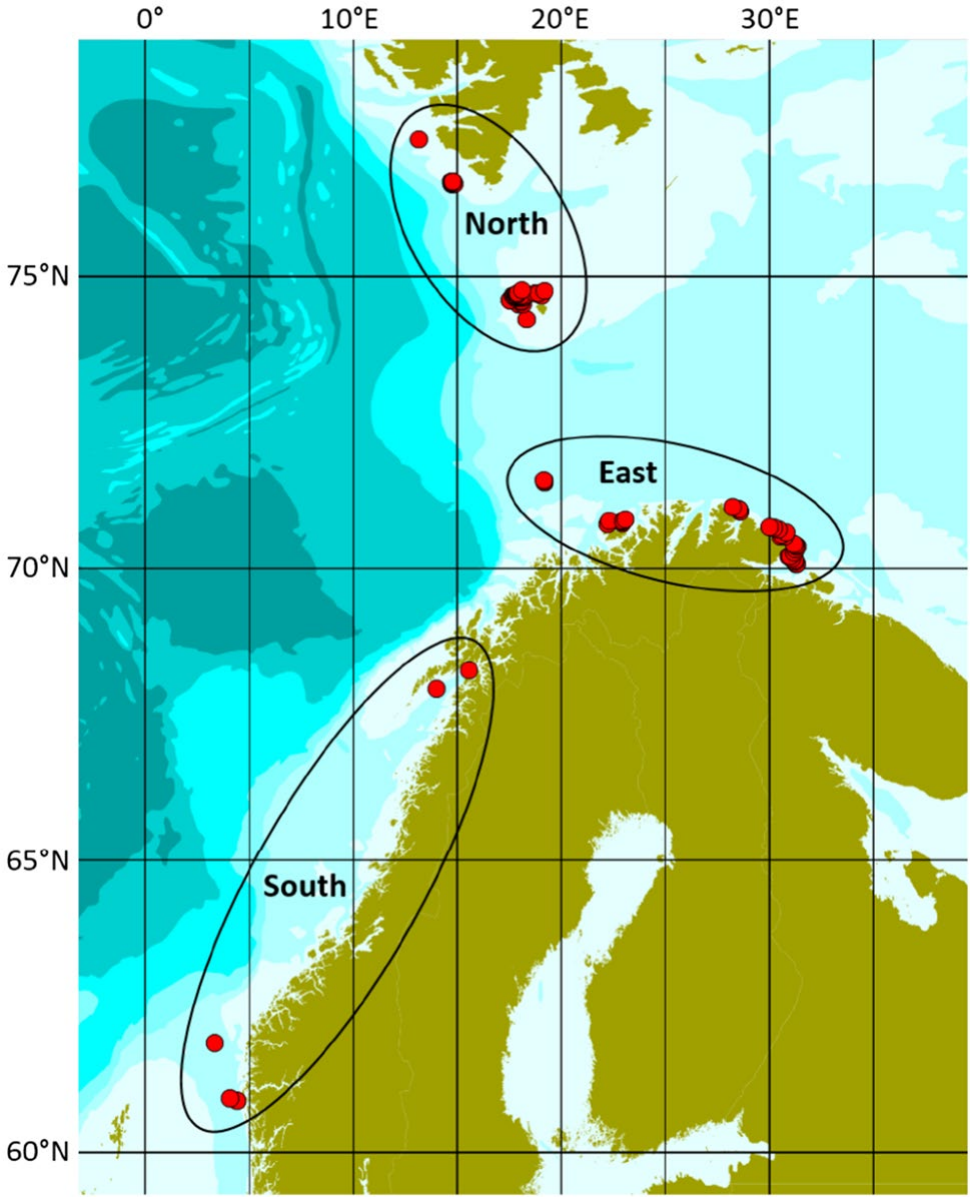
Map of northern Scandinavia showing the different sampling localities and areas

Whale meat samples were collected from nine different whaling boats between 1 May and 16 August, 2011, in the North East Atlantic. The geographical distribution of the sampling localities is given in Fig. 1. A total of 84 individuals were collected from three areas: (1) area North; including 46 whales caught near Svalbard and near Bear Island (74°16′N–77°21′N and 13°09′E–19°10′E), (2) area East; including 33 whales caught along the coast of northeastern Norway (70°05′N–71°30′N and 19°09′E–31°19′E), and (3) area South; including five whales from coastal Norway south of 69°N (two whales from the area 67°57′N–68°16′N and 14°00′E–15°33′E and three whales from the area 60°53′N–61°52′N and 03°21′E–04°24′E).

Each sample was placed into individual plastic bags and kept at −20°C until the boat came to harbor. The sample was then sent overnight to our laboratory. The samples were then freeze dried, water content was determined, and further homogenized and stored in tight plastic containers until analysis. The meat samples were analyzed for THg, MeHg, TAs, iAs, Cd, Pb and Se.

For the total element analyses, two weighed samples of 0.2–0.26 g were digested using 2.0 mL of concentrated nitric acid and 0.5 mL 30% (w/w) of hydrogen peroxide in a Milestone-MLS-1200 microwave oven (Milestone Inc., Shelton, CT, USA) as described by Julshamn et al. (2007). The measurements were performed by use of an Agilent 7500c ICP-MS (Agilent Technologies, Santa Clara, CA, USA). Results were reported as the average of two analytical parallels as required for the accreditation of the analysis. Agreement between parallels were required for the acceptance of the analytical results. A random selection of 44 out of the 84 samples were analyzed for MeHg. The method for determining MeHg comprised of spiking the tissue sample with a Me^201^Hg enriched spike solution followed by digestion of the sample with tetramethylammonium hydroxide (TMAH), adjustment of pH values by NaAc/HNO_3_, derivatisation with NaBEt_4_, and organic extraction of the derivatized EtMeHg into hexane. The hexane layer was subsequently analyzed by GC–ICP–MS (Agilent Technologies), and the result calculated using the isotope dilution equation (Valdersnes et al. 2012). Twenty samples of whale meat were selected and analyzed for iAs. Freeze dried material was weighed, 0.9 M NaOH in 50% (v/v) ethanol was added and the sample was extracted in CEM MARS 5 microwave oven using GreenChem Plus Teflon bombs for 20 min at 90°C (CEM Corp., Matthews, NC, USA). The samples were cooled, centrifuged and filtered. The iAs was determined using HPLC–ICP–MS (Agilent Technologies) fitted with an ICSep ION-120 ion exchange column (Transgenomic Inc, Omaha, NE, USA). All methods are accredited by the Norwegian Accreditation Authority (Lillestrøm, NO). Quality control was done by concomitant analyses of certified reference materials (TORT-2; National Research Council, Ottawa, ON, Canada). Our results in mg/kg for the TORT-2 were as follows (with the certified values in parentheses): THg 0.28 (0.27 ± 0.06), MeHg 0.160 (0.152 ± 0.013), TAs 22.4 (21.6 ± 1.8), Cd 27.1 (26.7 ± 0.6), Pb 0.33 (0.35 ± 0.13). At the time of analysis no reference materials were available for iAs, so TORT-2 was spiked with 50 ng of both As(III) and As(V), and recoveries were found to be in the range 96%–102%. The limits of quantification (LOQ) were 0.005, 0.03, 0.03, 0.013, 0.005, 0.03 and 0.01 mg/kg dry weight for THg, MeHg, TAs, iAs, Cd, Pb and Se, respectively.

Since THg, Cd, TAs and Se concentrations were skewed with long tails of high values, and displayed heteroscedasticity, the concentrations were log-transformed to remedy this. For comparison of physical parameters and element concentrations between different areas and between male and female whales, one-way analysis of variance (ANOVA) followed by Tukey’s multiple comparison test were used. Linear regressions were carried out using both log-transformed and untransformed concentrations. Since both analyses gave the same conclusions, results from untransformed concentrations are reported. Dell Statistica (Dell Inc., Round Rock, TX, USA), version 13, was used for statistical analyses and graphs, and a significance level of 0.05 was selected for all analyses.

## Results and Discussion

Length of the whales ranged from 525 to 910 cm, with a mean/median of 719/730 cm (Table 1). Seven males, 75 females and two whales with unknown sex were collected. A test for difference revealed that the dataset had significantly more females than males (*p* < 0.0001). There was no significant difference in length between female and male whales or between whales from the different geographical areas (i.e. North, East and South).

**Table 1:** Length of whales (cm) and concentration of TAs, Cd, THg, Pb, Se and MeHg (mg/kg w.w.) in meat samples of whales from different geographical areas

	North (N = 46) Mean ± SD Median (min–max)	East (N = 33) Mean ± SD Median (min–max)	South (N = 5) Mean ± SD Median (min–max)	Overall (N = 84) Mean ± SD Median (min–max)
Length	726 ± 91 735 (536–880)	716 ± 94 717 (530–910)	683 ± 140 740 (525–810)	719 ± 95 730 (525–910)
TAs	0.29 ± 0.12^a^ 0.25 (0.13–0.65)	0.19 ± 0.08^b^ 0.17 (0.084–0.44)	0.28 ± 0.12^a^ 0.22 (0.18–0.44)	0.25 ± 0.12 0.21 (0.084–0.65)
Cd	0.009 ± 0.008 0.006 (0.002–0.036)	0.007 ± 0.007 0.004 (0.002–0.031)	0.010 ± 0.009 0.007 (0.002–0.025)	0.008 ± 0.008 0.005 (0.002–0.036)
THg	0.14 ± 0.09 0.12 (0.05–0.49)	0.16 ± 0.09 0.13 (0.05–0.43)	0.20 ± 0.10 0.15 (0.11–0.35)	0.15 ± 0.09 0.12 (0.05–0.49)
Pb^d^	0.014 ± 0.012 0.009 (23)^c^ (<0.007–0.065)	0.012 ± 0.014 0.008 (18)^c^ (<0.006–0.089)	0.016 ± 0.016 0.010 (3)^c^ (<0.008–0.045)	0.013 ± 0.013 0.009 (44)^c^ (<0.006–0.089)
Se	0.22 ± 0.07^a^ 0.19 (0.15–0.49)	0.22 ± 0.05^a^ 0.21 (0.15–0.40)	0.29 ± 0.05^b^ 0.28 (0.23–0.37)	0.22 ± 0.06 0.20 (0.15–0.49)
MeHg	N = 30 0.14 ± 0.09 0.12 (0.05–0.48)	N = 10 0.19 ± 0.12 0.19 (0.06–0.45)	N = 4 0.23 ± 0.11 0.21 (0.14–0.36)	N = 44 0.16 ± 0.10 0.14 (0.05–0.48)

Letters “a” and “b” indicate significant differences (*p* < 0.05)

^c^The numbers in parentheses are the number of whale samples with Pb concentrations above LOQ

^d^Upperbound concentrations, calculated on the assumption that all the values below the limit of quantification are equal to the limit of quantification, were used for calculations of Pb

The concentrations of THg, MeHg, Cd, Pb, TAs and Se in the whale meat samples are shown in Table 1. Mean, median, minimum and maximum values are shown for each geographical area and overall. A comparison of element concentrations between female and male whales revealed no significant differences, but this result must be interpreted with caution since <10% of the whales were male. For THg, MeHg, Cd and Pb, we found no significant differences in the concentrations between the three different geographical areas, North, East and South. For Se, however, the concentration was significantly higher in the area South compared to both areas North and East (*p* < 0.05). For TAs the levels were higher in whales from the area North compared to the area East (*p* < 0.0005). Also, the concentration of TAs in whales from the area South was higher than in the East, but this difference was not significant, which can be explained by the low number of whales in the area South. It is conceivable that differences in Se and TAs concentrations in whales from different geographical areas may reflect differences in TAs content of the prey items available in different areas. Haug et al. (2002) have found that krill is the dominant prey for minke whales in the northern Barents Sea, whereas young Norwegian spring spawning (NSS) herring is the main food for minke whales from the southern regions of the Barents Sea. This difference in prey items cannot explain, however, the difference in TAs concentration between the area North and East in this study, since krill and NSS-herring typically contain similar low levels of TAs (Edmonds and Francesconi 2003; Frantzen et al. 2015; Julshamn et al. 2012).

The concentration of THg in the meat samples of individual whales varied between 0.05 and 0.49 mg/kg wet weight (w.w.) (Table 1), and no samples had values of THg above the maximum level of 0.5 mg/kg w.w. established by the European Union (EU) for muscle meat of fish for human consumption (EU 2006). No maximum levels for THg or other elements in whale meat have been established in EU, but in Japan a maximum level of 0.4 mg/kg w.w. has been set for THg in whales (JMHLW 1973). Four whales (4.8%) exceeded this Japanese limit, two from the area North and two from the area East. Since MeHg is the most toxic Hg compound when assessing human exposure through food (Dietz et al. 2013), we also measured MeHg and found that 100% of the Hg in the meat of minke whale was MeHg (Table 1). Japan has also established a maximum limit for MeHg of 0.3 mg Hg/kg w.w., and three (6.8%) of the 44 samples analyzed for MeHg exceeded this limit. All samples were below the guideline levels for MeHg in fish of 0.5 mg/kg set by the Codex Alimentarius Commission (CAC 2016). The mean concentration of THg in our study, 0.15 mg/kg w.w. (Table 1) was slightly lower than earlier results from the Norwegian Veterinary Institute that analyzed 64 pooled samples and 61 individual samples of minke whale from the same area in 2002. They found a mean of 0.23 mg Hg/kg w.w. (Kleivane and Børsum 2003). However, our results were slightly higher than results found in minke whale meat from the Northern Pacific Ocean in samples bought from the Japanese market (Endo et al. 2003). They reported mean concentrations of THg of around 0.10 mg Hg/kg w.w. Further, they reported mean concentrations as low as 0.03 mg Hg/kg in Antarctic minke whale (*Balaenoptera bonaerensis*). The high percentage of MeHg found in our study is in line with the majority of the results found previously on whale meat from the Japanese market (Endo et al. 2005). In comparison with odontocete whales, the mysticete whales, such as minke whale, have much lower Hg levels. Typical values for meat of pilot whales (*Globicephala melas*) in the Northern Atlantic have been reported to be around 2 mg Hg/kg w.w. (Dam and Bloch 2000). The reason for the lower levels of Hg in minke whale compared to pilot whale, is probably that the minke whale feed on prey with rather low Hg levels such as herring and krill in the north (Frantzen et al. 2015; Haug et al. 2002) and sand eel further south (Pierce et al. 2004).

We found no correlation between the THg concentration and the length of the whales, neither for the whole dataset nor for the individual geographical areas (results not shown). This is in contrast to results from various fish species showing increasing Hg concentrations with increasing age and size of the fish (Frantzen et al. 2015; Julshamn et al. 2013). Positive correlations of Hg with length, age and weight have also previously been documented for odontocete whales, but for these whales the correlation with Hg was stronger in liver than in muscle and stronger for age than for length and weight (Honda et al. 1983).

The whale meat samples in this study had low, but detectable levels of Cd (Table 1) with a mean/median concentration of 0.008/0.005 mg/kg w.w. No samples had Cd concentrations above 0.05 mg/kg w.w., which is the EU maximum level for Cd in fish muscle for human consumption (EU 2006). Also for Pb, the levels were low, below or close to the quantification limit of the method for most of the samples. An upperbound mean/median level of 0.013/0.009 mg Pb/kg w.w. was found, i.e. much lower than the EU maximum level for Pb in fish muscle of 0.3 mg/kg w.w. (Table 1). The TAs content in minke whale meat varied between 0.08 and 0.65 mg/kg w.w. with a mean/median concentration of 0.25/0.21 mg/kg w.w. This is lower than in muscle from most fish species where it is common to observe mean TAs levels higher than 1.0 mg /kg w.w. (Julshamn et al. 2012). In liver samples from pilot whale and beluga whale (*Deliphinapterus leucus*), As concentrations in the range of 0.17–1.27 mg/kg w.w. were reported, but As levels in the meat of these whales were not determined (Goessler et al. 1998). In our study, 20 whale meat samples were also analyzed for iAs, the most toxic form of As, and the results revealed that the levels in all samples were below the method LOQ of 0.003 mg As/kg w.w. We found no correlation between the levels of TAs or Pb and length of the whales, neither when looking at the overall dataset nor when analyzing the individual areas separately. For Cd we found a positive correlation with length, which was significant for the overall dataset (*p* < 0.00001) and for the area North (*p* < 0.0005) and East (*p* < 0.005), but not South. The lack of significant correlation between Cd-concentration and length for the area South, is probably due to the low number of whales in this area. Positive correlations of Cd and Pb with age, length and weight have previously been shown for dolphins, but the levels of Cd and Pb in the dolphins were higher and the correlations were stronger for age than for weight and length, as well as stronger for Cd than for Pb for these odontocete whales (Honda et al. 1983).

The Se concentration in the whale meat varied between 0.06 and 0.49 mg/kg w.w. with a mean/median level of 0.22/0.20 mg/kg w.w. This level is comparable to levels found in lean fish muscle (NIFES 2017), but lower than levels found in odontocete whales (Endo et al. 2005). There was no correlation between the levels of Se and length of the whales when the overall dataset or whales from the area North and South were analyzed, but a significant negative correlation was found for whales from the area East (*p* < 0.05).

**Fig. 2:**
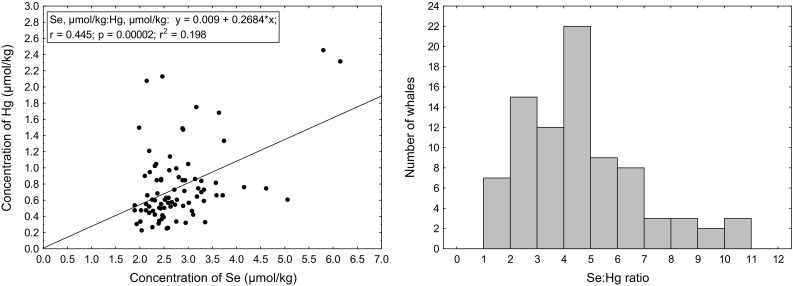
Regression analysis of Se versus Hg (*left*) and histogram of Se:Hg ratio (*right*)

Se is suggested to have a protective role against the toxicity of Hg (Ralston and Raymond 2010). Linear regression analysis showed a significant positive correlation between Se and Hg (*p* < 0.00005), but there was a large variation between individual whales (Fig. 2, left). The Se:Hg molar ratio was found to be in the range of 1.0–10.3 for individual whales with a mean/median of 4.6/4.4 (Fig. 2, right), showing an ample surplus of Se to sequester the Hg levels present. This molar excess of Se compared to Hg, is typical to what has been found in many other fish species and in dolphin (Burger and Gochfeld 2012).

In conclusion, there was little or no variation in element concentrations between whales from different geographical areas, and no difference was found between the concentrations in female and male whales. The concentration of Cd increased with increasing length of the whales, but no correlation was found between the levels of THg, TAs or Pb and the length of the whales. Meat of minke whale contained low levels of Cd, Pb, TAs and iAs, which posed no risk from a seafood safety perspective. Moderate levels of Hg were found in the whale meat samples, and all the Hg in meat was in the form of MeHg. However, the whale meat also contained substantial amounts of Se, which is proposed to reduce harmful effects of high Hg intake. For a 60 kg person, consumption of 100 g of whale meat with the highest concentration of Hg (i.e. MeHg) found in this study (0.49 mg/kg w.w.) would lead to an intake corresponding to 51% of the provisional tolerable weekly intake (PTWI) set by JECFA (1.6 µg/kg bw) or 63% of the tolerable weekly intake (TWI) set by EFSA (1.3 µg/kg bw) (EFSA 2012; JECFA 2004).
